# The dual role of sirtuins in cancer: biological functions and implications

**DOI:** 10.3389/fonc.2024.1384928

**Published:** 2024-06-14

**Authors:** Lu Yu, Yanjiao Li, Siyuan Song, Yalin Zhang, Yiping Wang, Hailian Wang, Zhengteng Yang, Yi Wang

**Affiliations:** ^1^ Department of Respiratory, Sichuan Academy of Medical Sciences and Sichuan Provincial People’s Hospital, School of Medicine, University of Electronic Science and Technology of China, Chengdu, China; ^2^ Department of Pharmacy, Qionglai Hospital of Traditional Chinese Medicine, Chengdu, China; ^3^ Department of Neuroscience, Baylor College of Medicine, Houston, TX, United States; ^4^ School of Medicine, University of Electronic Science and Technology of China, Center of Critical Care Medicine, Sichuan Academy of Medical Sciences, Chengdu, China; ^5^ Center of Critical Care Medicine, Sichuan Academy of Medical Sciences and Sichuan Provincial People’s Hospital, School of Medicine, University of Electronic Science and Technology of China, Chengdu, China; ^6^ Clinical Immunology Translational Medicine Key Laboratory of Sichuan Province, Center of Organ Transplantation, Sichuan Academy of Medical Science, Nanning, China; ^7^ Department of Medicine, The First Affiliated Hospital of Guangxi University of Traditional Medicine, Nanning, China

**Keywords:** sirtuins, histone deacetylase, metabolic functions, tumor, cancer therapy

## Abstract

Sirtuins are pivotal in orchestrating numerous cellular pathways, critically influencing cell metabolism, DNA repair, aging processes, and oxidative stress. In recent years, the involvement of sirtuins in tumor biology has garnered substantial attention, with a growing body of evidence underscoring their regulatory roles in various aberrant cellular processes within tumor environments. This article delves into the sirtuin family and its biological functions, shedding light on their dual roles—either as promoters or inhibitors—in various cancers including oral, breast, hepatocellular, lung, and gastric cancers. It further explores potential anti-tumor agents targeting sirtuins, unraveling the complex interplay between sirtuins, miRNAs, and chemotherapeutic drugs. The dual roles of sirtuins in cancer biology reflect the complexity of targeting these enzymes but also highlight the immense therapeutic potential. These advancements hold significant promise for enhancing clinical outcomes, marking a pivotal step forward in the ongoing battle against cancer.

## 1 Introduction

Epigenetics, encompassing DNA modifications, histone alterations, and chromatin remodeling, has emerged as a focal point in oncological research over recent years (1–3). Among the myriad epigenetic mechanisms, the acetylation and deacetylation of histones stand out as a pivotal mode of covalent histone modification (4, 5). Central to this process are the Sirtuin (Sirt) enzymes (6, 7). Originating from the discovery of the Sir2 gene in yeast, Frye identified five human homologs in 1999, dubbed Sirt1 through Sirt5. Subsequent research expanded the family with the cloning of Sirt6 and Sirt7 from a human spleen cDNA library (8). The sirtuin family’s extensive involvement in a range of biological functions and pathologies, particularly cancer, underscores their significance. Sirtuins critically modulate the metabolic adaptations of cancer cells that favor growth and division and are implicated in the dysregulation of DNA repair and apoptotic pathways that contribute to genomic instability, mutation accumulation, and ultimately, tumor development and progression (9, 10). The intricate role of sirtuins extends to key cellular processes such as the cell cycle, DNA repair, cell survival, and apoptosis, positioning them as pivotal players in the cellular response to genomic instability and tumor dynamics. Given their broad biological impact, sirtuins and their inhibitors have become focal points in the quest for novel cancer therapies. Investigations have shown that sirtuins regulate Base Excision Repair (BER), Nucleotide Excision Repair (NER), and DNA double-strand break (DSB) repair pathways, with Sirt1, in particular, controlling Forkhead Box O (FoxO) protein deacetylation and apoptosis induction (11, 12). SIRT3 localizes mainly to the mitochondrial matrix and plays an important role in regulating mitochondrial metabolism, including the tricarboxylic acid (TCA) cycle, the urea cycle, amino acid metabolism, fatty acid oxidation, ETC/oxidative phosphorylation (OXPHOS), ROS detoxification, mitochondrial dynamics and the mitochondrial unfolded protein response (13).The regulation of various signaling pathways by sirtuins through acetylation processes is crucial in tumor biology, influencing outcomes such as tumor initiation, metastasis, and angiogenesis. The differential effects of sirtuin mutations, overexpression, up-regulation, and activation on cancer further underscore the complexity of their roles (9, 10).

Given the pivotal function of histone deacetylases like sirtuins in the malignant progression of tumors, especially during invasion and metastasis, their study offers critical insights for the development of new anti-cancer drugs and strategies for clinical cancer treatment.

## 2 Comprehensive overview of the sirtuin family: from molecular functions to therapeutic potential

**Figure 1:**
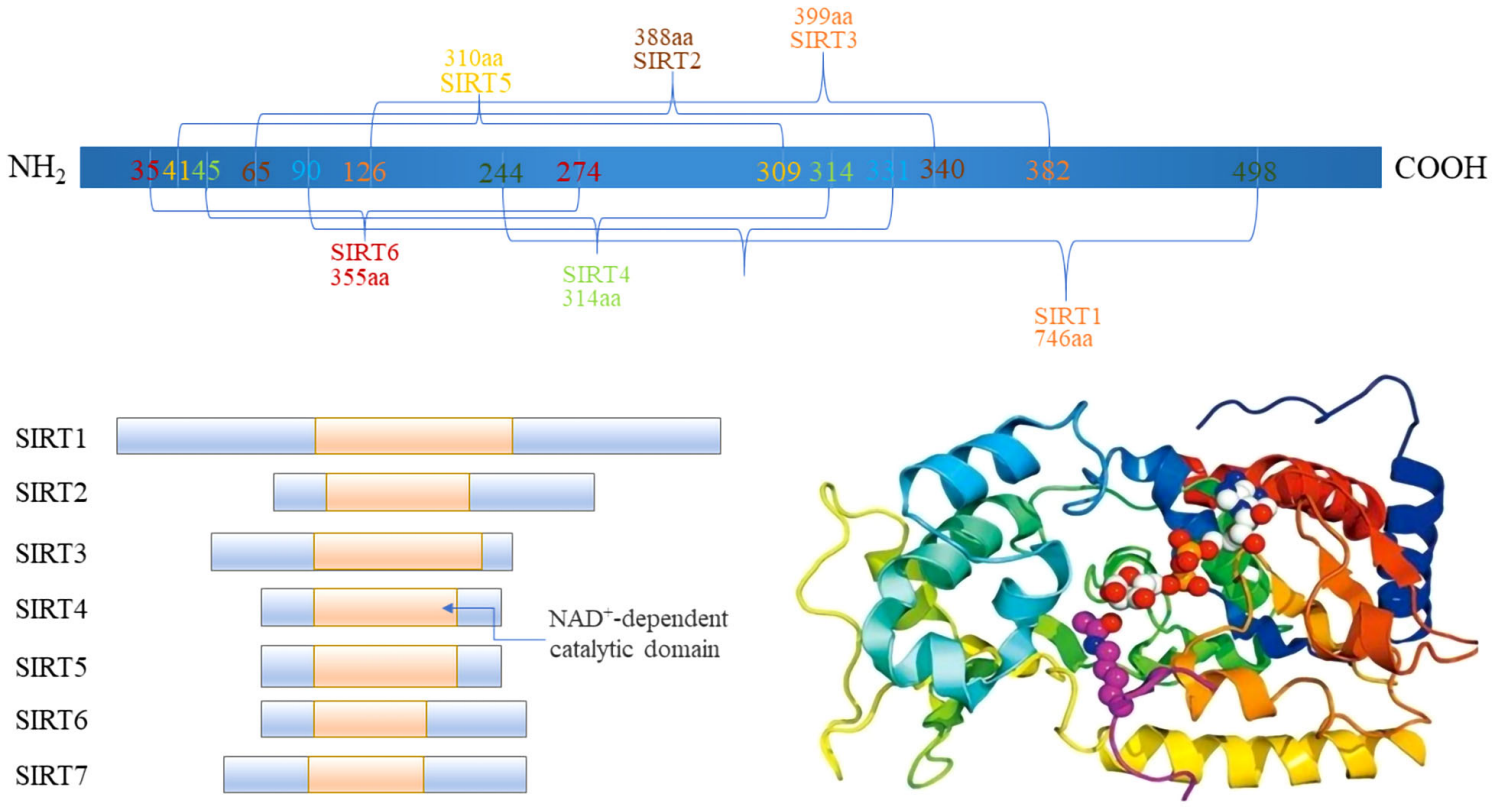
The Domain of Sirtuins.

The Sirtuin family, integral to class III histone deacetylase (HDACs), exhibits inhibition by nicotinamide and spans a broad evolutionary spectrum from bacteria to humans. Characterized by a conserved catalytic core, sirtuins engage in the deacetylation of diverse substrates. In mammals, this family comprises seven distinct members, SIRT1–7, each occupying unique genetic loci across chromosomes 10q22.1, 19q13, 11p15, 5, 12q, 5p23, 19p13.3, and 17q25, with gene lengths varying from 6 to 37 (14, 15) (**Figure 1**). These sirtuins possess specific domains, underlining their structural and functional diversity.

A typical sirtuin structure includes about 270 amino acids, featuring a large domain with Rossmann folds and a smaller, less conserved domain with a zinc ribbon and helical segments, facilitating the catalytic process (16). Subcellular localization differentiates sirtuins’ functions (**Table 1**): SIRT1 is mainly nuclear, but a cytoplasmic localization has also been reported (17). Heterokaryon assay shows that SIRT1 has nucleocytoplasmic shuttle phenomenon (18). SIRT6, and SIRT7 are nuclear. SIRT6 is mainly found in chromatin and SIRT7 is mainly found in nucleoli, regulating genetic and metabolic activities (19, 20). SIRT2 is cytoplasmic, but it is also found in the nucleus of the G2 to M phase transition of the cell cycle (21). SIRT1 involves a variety of physiological and pathological processes, including cell proliferation, apoptosis, DNA repair, metabolic regulation, and antioxidant defense. It is also involved in the regulation of the aging process and longevity (22). SIRT2 regulates microtubule stability and cell cycle progression. In addition, SIRT2 has also been implicated in the pathogenesis of neurodegenerative diseases (23). SIRT3, SIRT4, and SIRT5 are mitochondrial, overseeing oxygen and energy supply (24). SIRT3 promotes cell metabolism and deacetylates specific substrates to play a carcinogenic role. SIRT3 plays a unique function by mediating interactions between mitochondria and intracellular signaling. SIRT3 regulates oxidative stress, amino acid metabolism, fatty acid oxidation, electron transport, and the TCA cycle through deacetylating various substrate proteins (25). Beyond their deacetylase activity, SIRT4–6 exhibit additional enzymatic functions. In addition to their deacetylase activities, the sirtuins SIRT4–6 possess unique enzymatic functions that are crucial for cellular metabolism and stress responses. SIRT4, for example, has strong Adenosine diphosphate (ADP)-ribosyltransferase activity, allowing it to modify other proteins and influence cellular processes such as insulin secretion and amino acid catabolism (26). SIRT5 distinguishes itself by targeting and removing malonyl and succinyl groups from proteins, which regulates the urea cycle and mitochondrial function, demonstrating its critical role in metabolic regulation (27). SIRT6, on the other hand, is involved in DNA repair, telomere maintenance, and also has deacetylase and long-chain deacylase activity, making it a pivotal player in genomic stability and aging (28). These multifaceted activities underscore the sirtuins’ crucial roles in not just metabolism and energy production, but also in maintaining cellular homeostasis under stress conditions, providing potential targets for therapeutic interventions in metabolic disorders and age-related diseases.

**Table 1:** Classification of SIRTs including localization, enzymatic activity, targets, and function.

Sirtuin	Localization	Enzymatic activity	Histone deacetylation target	Non-histone deacetylation target	Function	References
SIRT1	Nucleus	Deacetylase	H1-K26Ac H3-K9Ac H4-K16Ac H4-K20me H3-K9me3	HIF-1α HIF-2α MYC	Glucose Metabolism, Differentiation, Neuroprotection, Insulin secretion	(15)
SIRT2	Cytoplasm	Deacetylase	H3-K18Ac H3-K56Ac H4-K16Ac	Tubulin Foxo3a EIF5A P53 G6PD MYC	Cell-cycle control, Tubulin deacetylation	(28, 29)
SIRT3	Mitochondria	Deacetylase	H3K56ac H4K14 ac	SOD2 PDMC1a IDH2 GOT2 FoxO3a	ATP-production, Regulation of mitochondrial proteins deacetylation, Fatty-acid oxidation	(33, 35–38)
SIRT4	Mitochondria	ADP-ribosylase		GDH PDH	Insulin secretion	(24, 39, 40)
SIRT5	Mitochondria	Malonyl-Succinyl- Glutaryl- Deacetylase		CPS1	Urea cycle	(25, 41, 42)
SIRT6	Nucleus	Deacetylase, ADP-ribosyltransferase, Long-chain fatty acyl Deacylase	H3K9ac H3K56ac H3-K18Ac		Telomeres and Telomeric functions, DNA repair	(17, 26, 28, 29, 43–46)
SIRT7	Nucleus	Deacetylase	H3-K18Ac	HIF-1α HIF-2α	RNA pol I transcription	(17, 43–46)

Sirtuins intersect with multiple biological pathways, engaging with proteins such as Tumor protein p53 (P53), FOXO/Peroxisome proliferator-activated receptor-γ coactivator-1 (PGC-1), Nuclear Factor Kappa B (NF-kB), and Ku70 to modulate stress responses, metabolism, aging, and apoptosis. Their pivotal role in cellular processes extends to DNA repair, oxidative stress, and aging (29). Given their critical involvement in tumorigenesis, sirtuins have attracted significant interest as potential targets in cancer research. The dual nature of SIRTs’ function, as either tumor suppressors or promoters, often depends on the cellular context and experimental conditions, with SIRT1 displaying bifunctionality and SIRT2 and SIRT6 predominantly acting as tumor suppressors (19, 30). Explorations into sirtuins’ roles have unveiled their potential in influencing the epigenome, offering insights into how these enzymes affect gene expression and cellular fate without altering the DNA sequence. This epigenetic regulation presents a nuanced layer of control over cellular processes, highlighting sirtuins’ significance in both health and disease states, particularly in cancer where they can dictate cell survival or death. Moreover, the therapeutic implications of targeting sirtuins in cancer treatment have gained momentum, driven by the understanding that modulating sirtuin activity can alter the trajectory of cancer progression. As research delves deeper into the mechanistic pathways governed by sirtuins, their capacity to serve as biomarkers for cancer prognosis or as direct targets for novel anticancer therapies becomes increasingly apparent. This burgeoning area of study not only promises to enhance our grasp of cancer biology but also to usher in a new era of targeted treatments that leverage the unique properties of the sirtuin family.

## 3 Sirtuin proteins: guardians of metabolic and genomic stability

### 3.1 Sirtuin proteins and their metabolic functions

Sirtuin proteins, commonly referred to as SIRTs, are essential components in the regulation of cellular metabolism, impacting the processing of sugars and fats at various biological levels. These enzymes are pivotal in the management of critical metabolic pathways, such as those involved in the metabolism of glucose—encompassing aerobic glycolysis, the tricarboxylic acid (TCA) cycle, and glutamine metabolism—and fat metabolism, which includes the breakdown of fatty acids, cholesterol, and the production of ketone bodies. Such metabolic activities are crucial in the pathogenesis and progression of cancerous tumors (31). Sirtuins exert their regulatory influence over these metabolic processes by modulating the activity of vital enzymes and transport systems through mechanisms that include transcriptional and post-translational modifications, enzyme function adjustments, and the manipulation of signaling pathways.

**Figure 2:**
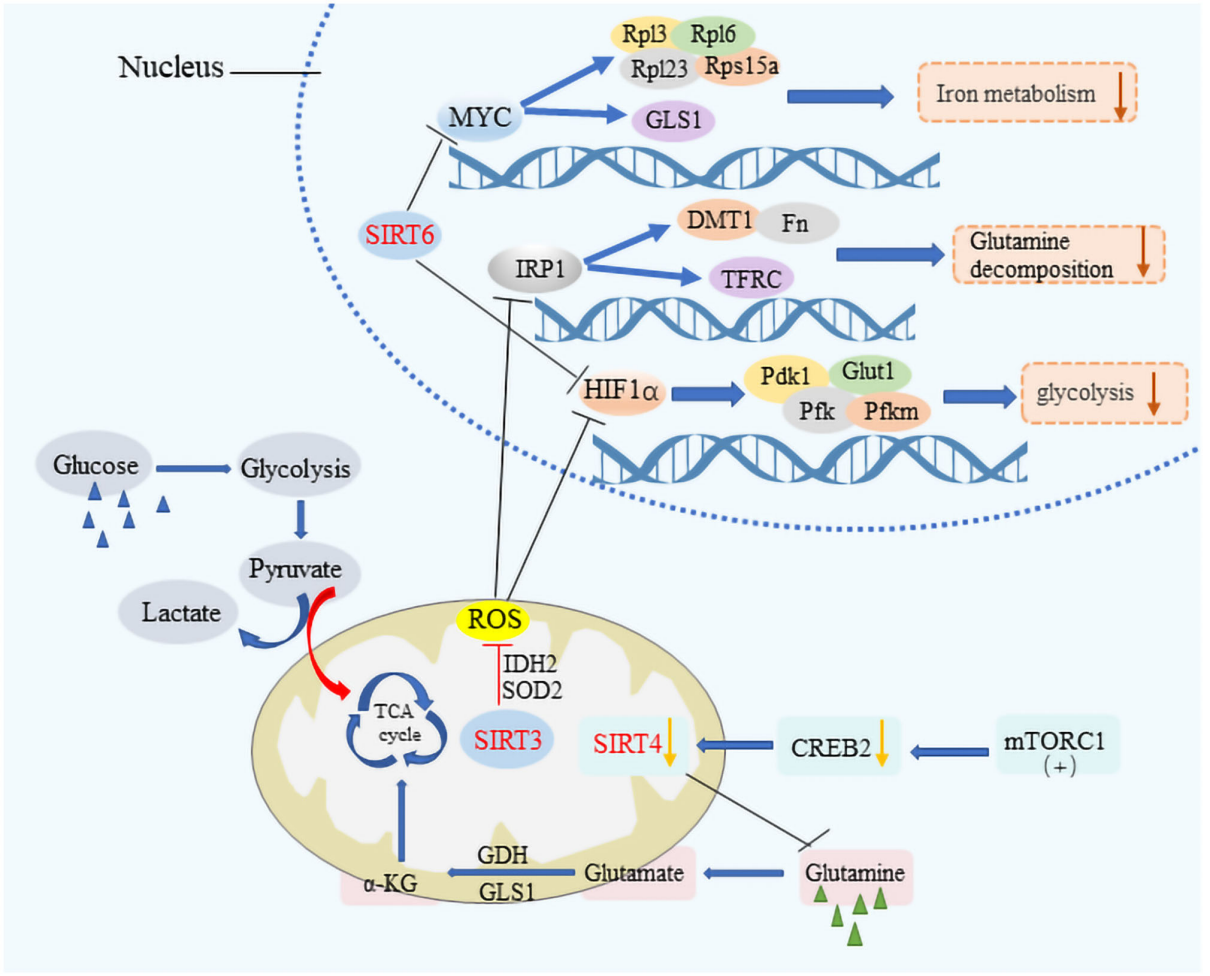
The metabolic modulation by SIRTs in cancer cells. SIRT3, SIRT4, and SIRT6 significantly impact cancer cell metabolism by downregulating key pathways crucial for tumor growth. These sirtuins suppress HIF-1α and IRP1 target genes, affecting glycolytic processes by inhibiting genes such as PDK1, PFK, and Pfkm, alongside Glut1, which is vital for glucose transport. They also regulate iron metabolism by modulating the expression of DMT1 and other bivalent metal transporters, as well as ferritin and the transferrin receptor. The influence extends to MYC target genes, including ribosomal proteins Rpl3, Rpl6, Rpl23, and Rps15a, thereby reducing the metabolic rate encompassing aerobic glycolysis, the tricarboxylic acid (TCA) cycle, and glutamine decomposition. Additionally, the activation of the mTORC1 pathway leads to CREB2 degradation, which results in decreased SIRT4 expression, illustrating a comprehensive mechanism by which these sirtuins suppress metabolic pathways to hinder cancer progression.

SIRT1, for instance, is known to enhance the expression of FOXO1 and PGC-1α or to suppress the activity of transcription factors like sterol regulatory element binding protein-1c (SREBP1c), thereby reducing the cellular fat content by lowering the expression of genes targeted by SREBP1c (32–35). This action underscores SIRT1’s significant role in maintaining metabolic health across various tissues, including muscle, liver, and fat tissue, through the deacetylation of numerous target proteins. Similarly, SIRT3 is instrumental in metabolic regulation by deacetylating and activating key enzymes such as acetyl-CoA synthetase 2 (ACSS2) and glutamate dehydrogenase (GDH), thus facilitating an enhanced Krebs cycle (36). It also plays a role in managing cellular reactive oxygen species (ROS) levels and mitigating ROS-induced activation of iron regulatory protein 1 (IRP 1), which in turn moderates glycolysis and glutamine breakdown (37) (**Figure 2**). The activity of SIRT3 is intricately linked to the metabolic state of the cell, modulated by the intracellular Nicotinamide Adenine Dinucleotide (NAD^+^)/Nicotinamide Adenine Dinucleotide Hydrogen (NADH) ratio (38–40). SIRT3 can maintain mitochondrial homeostasis, which is necessary for survival Therefore, SIRT3 is regulated in numerous ways. As a major repair enzyme in the base excision repair pathway, 8-oxoguanine DNA glycosylase (OGG1) is a newly discovered target of SIRT3. Cheng et al. found that SIRT3 deacetylates OGG1 to regulate its incision activity and decrease its degradation. Mitochondrial DNA damage repair, mitochondrial integrity, and apoptotic cell death induced by oxidative stress are closely related to the acetylation- and turnover-related regulation of OGG1 by SIRT3. SIRT3 can regulate different enzymes including manganese dismutase, SOD2, and catalase, which are important in regulating ROS levels (41). On the other hand, SIRT4, with its limited deacetylase but notable ADP-ribosyltransferase activity, modulates glutamine metabolism post-DNA damage and inhibits it through the ADP ribosylation of Glutamate Dehydrogenase (GDH). Furthermore, it has been discovered that mammalian target of rapamycin complex 1 (mTORC1) influences the expression of SIRT4 by promoting the degradation of its transcriptional activator, CAMP response element-binding protein 2 (CREB2), thereby affecting SIRT4 gene expression negatively (42, 43). SIRT5, possessing lysine deacetylase activity, plays a part in the regulation of glycolysis, the TCA cycle, fatty acid oxidation, and the formation of ketone bodies among other processes (44, 45). Additionally, SIRT6 and SIRT7, both of which exhibit ADP ribosyltransferase activity, contribute to the regulation of cellular metabolism with SIRT6 directly inhibiting the expression of various glycolysis-related genes and impacting iron metabolism (46–49). The reprogramming of cellular metabolism is a hallmark of cancer, with cancer cells adapting their metabolic processes to meet energy demands, a phenomenon in which sirtuins play a significant role by regulating metabolic pathways, thus influencing cancer initiation and progression (50–53).

### 3.2 Sirtuins and DNA repair mechanisms

**Figure 3:**
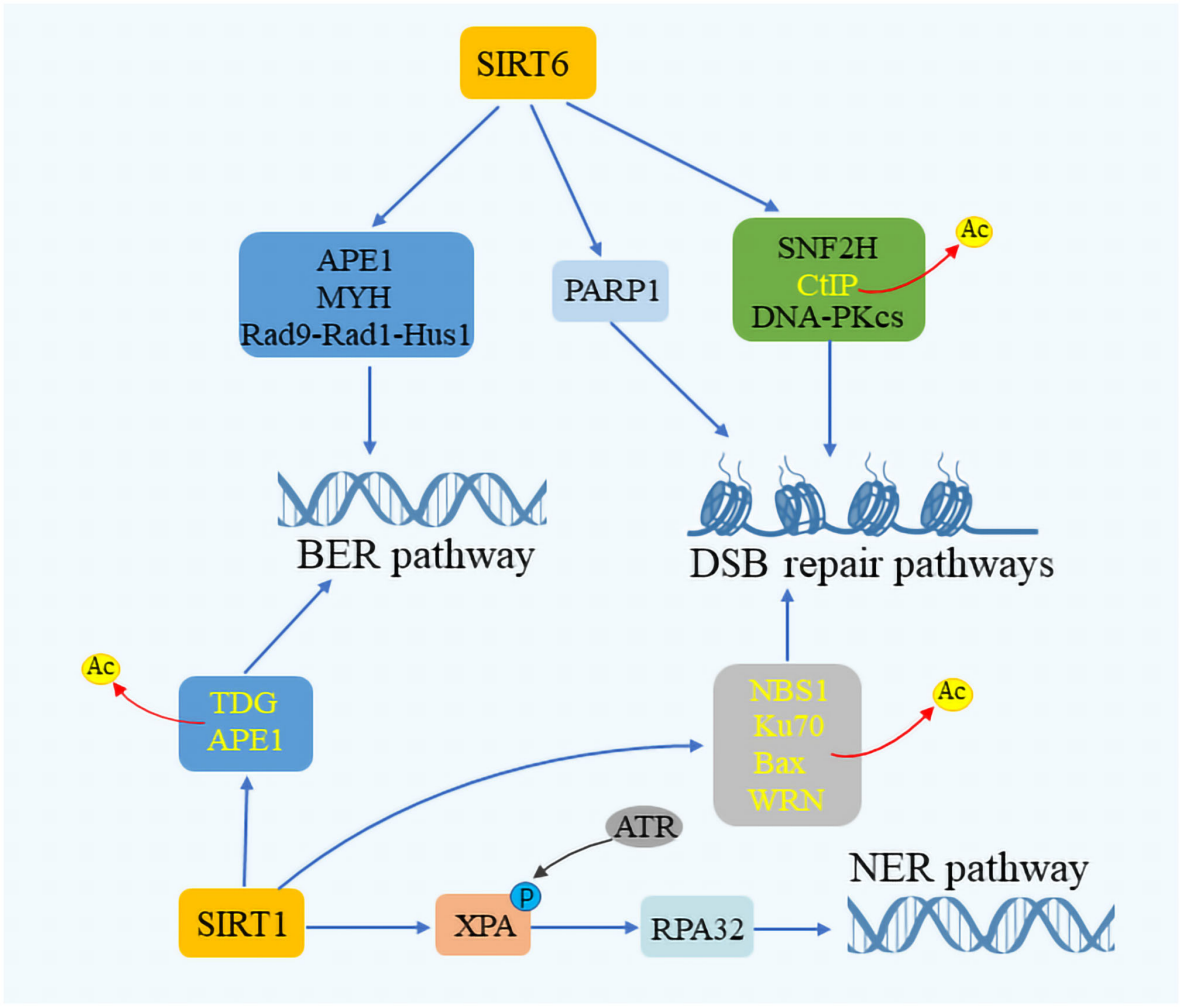
SIRTuins’ involvement in DNA repair mechanisms. SIRT6 plays a crucial role in the base excision repair (BER) pathway by interacting with key components such as Myosin heavy chain (MYH), Apurinic/Apyrimidinic endonuclease 1 (APE1), and the Rad 9-RAD 1-Hus 1 (9–1–1) complex. SIRT1 enhances the DNA repair process by promoting the deacetylation of APE1 and Thymine DNA glycosylase (TDG). In the context of double-strand break (DSB) repair, SIRT6 boosts the activity of ADP-Ribose polymerase 1 (PARP1) and facilitates the deacetylation of CtIP. It also interacts with the DNA-dependent protein kinase Catalytic subunit (DNA-PKcs) and Sucrose Nonfermenter2 Homolog (SNF2H), playing a vital role in responding to DNA damage. Moreover, SIRT1 is instrumental in augmenting DNA repair by promoting the acetylation of Nijmegen breakage syndrome 1 (NBS1), Ku70, Bax, and Werner syndrome protein (WRN). In the nucleotide excision repair (NER) pathway, SIRT1 increases the efficacy of DNA repair by enhancing the interaction between Rad3-related phosphorylation of Xeroderma Pigmentosum (XPA) and Replication protein A32 (RPA32), showcasing the comprehensive involvement of SIRTuins in maintaining genomic stability through various DNA repair mechanisms.

Sirtuins are critical for maintaining genome integrity, participating actively in the repair of DNA within both the nucleus and mitochondria. Their role in DNA repair involves the deacetylation of DNA repair components, thereby enhancing the efficiency of repair enzymes and improving the cellular response to damage (54–56). SIRT6, in particular, is highlighted for its pivotal role in DNA repair, with deficiencies in SIRT6 leading to genomic instability in mice (57). In cells under oxidative stress, SIRT6 promotes DSB repair via non-homologous end joining (NHEJ) and homologous recombination (HR) at DSB sites (58, 59). SIRT6 has been shown to form complexes with Myosin Heavy Chain (MYH), Apurinic/apyrimidinic Endonuclease 1 (APE1), and the 9–1-1 complex (RAD9-RAD1-HUS1 Checkpoint Clamp Component, HUS1), thus safeguarding genomic and telomere integrity and enhancing cellular resilience against oxidative stress (60) (**Figure 3**). Additionally, SIRT6 facilitates DSB repair by augmenting the activity of PARP1 and recruiting SNF2H at DNA damage sites for proper DSB repair (61). SIRT6’s interaction with CtIP and DNA-Protein kinase C (PKC) further underscores its role in DNA damage response (62, 63). SIRT1 also contributes to genome stability by deacetylating DNA damage repair factors, thereby facilitating the repair process. It enhances the BER pathway by promoting APE1 deacetylation and stimulating the activity of Thymine DNA glycosylase (TDG), which mediates DNA repair. In the NER pathway, SIRT1 plays a role by complementing XPA phosphorylation through interactions with ATR Serine/Threonine Kinase (ATR) and replication protein A 32 (RPA32), while in the DSB pathway, it aids in cell cycle regulation and DNA repair assessment through NBS1 deacetylation (64–68). Moreover, SIRT3 has been recognized for its role in repairing mitochondrial DNA (mtDNA) damage and preventing apoptotic cell death under oxidative stress conditions by regulating the acetylation and activity of 8-oxoG glycosylase1 (OGG1) (69).SIRT3 targets different enzymes which regulate mitochondrial metabolism and participate in ROS detoxification, such as the complexes of the respiratory chain, the isocitrate dehydrogenase, or the manganese superoxide dismutase (25). SIRT7 enhances DNA repair by boosting upstream signaling and inducing chromatin condensation, thus inhibiting transcription near DSB sites and limiting DSB mobility during the initial response phase (70, 71). This intricate network of sirtuin activities highlights the close relationship between sirtuin function and DNA damage repair, underscoring their vital role in maintaining cellular health and stability.

## 4 Sirtuins in managing cellular stress

**Figure 4:**
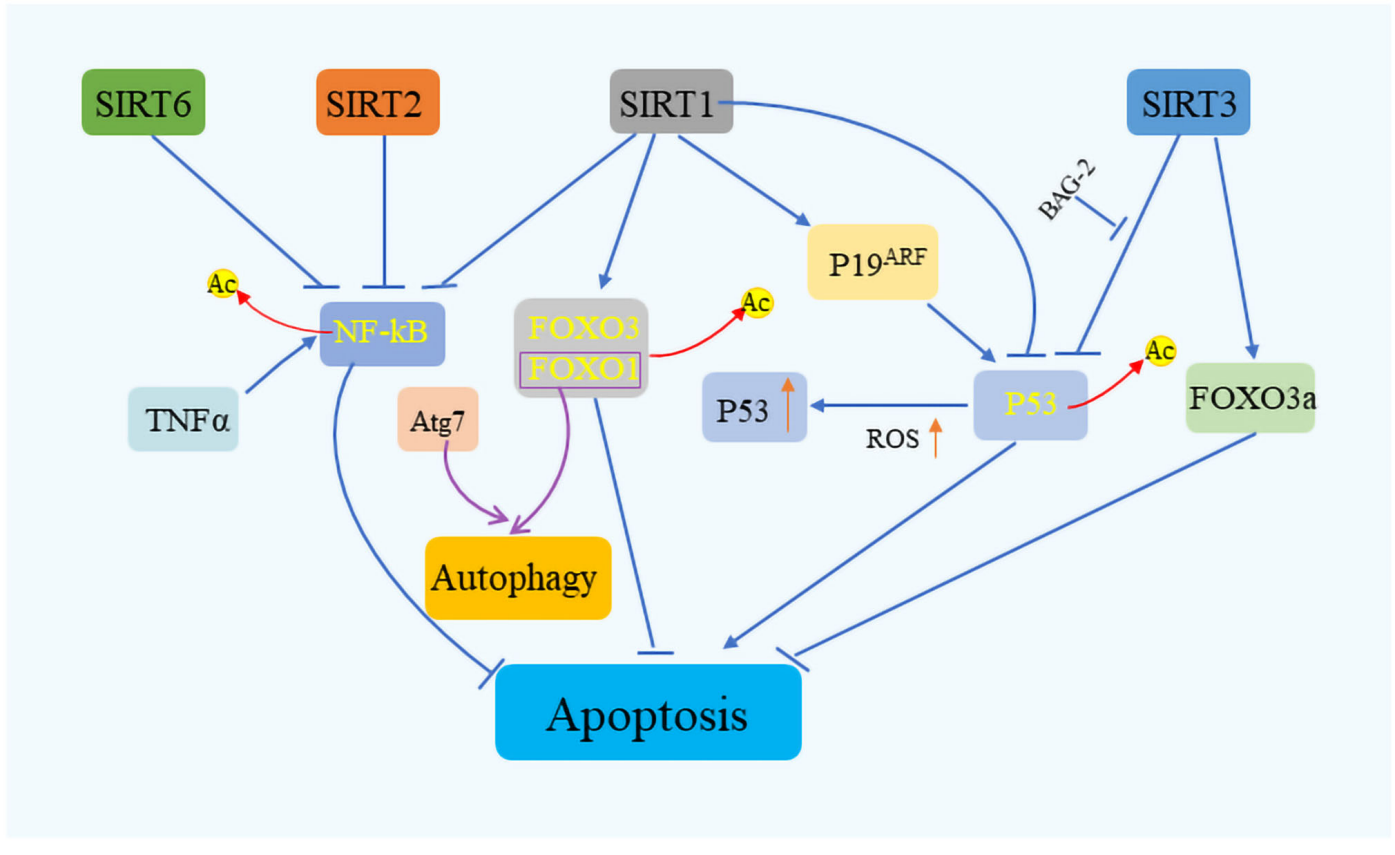
Sirtuins’ role in cell survival and stress response. Sirtuins orchestrate cell fate under various stress conditions by modulating key apoptotic and survival pathways. SIRT1 plays a pivotal role in cell survival by interacting with critical regulators such as P53, FOXO, and NF-κB, while SIRT2 and SIRT6 similarly influence NF-κB signaling. SIRT3, on the other hand, contributes to apoptosis regulation by targeting P53 and FOXO3a. Through these interactions, sirtuins serve as vital mediators of cellular stress response, ensuring cell survival by fine-tuning the balance.

The Sirtuin family of proteins plays a crucial role in sustaining cellular equilibrium in the face of stress. It has been documented that SIRT1 engages with stress-induced transcription factors including nuclear factor κB (NF-κB), heat shock transcription factor 1 (HSF1), and peroxisome proliferator-activated receptor gamma coactivator 1α (PGC-1α), exerting a regulatory influence (72–74). SIRT1’s interaction with these factors can both elevate cancer risk by hindering P53-induced apoptosis and, paradoxically, stimulate P53 activation through the modulation of P19 alternative reading frame (p19ARF), particularly under conditions of high intracellular ROS (75) (**Figure 4**). By promoting the deacetylation of P53, SIRT1 facilitates the accumulation of this protein in mitochondria, enhancing cellular survival mechanisms (76). Furthermore, SIRT1 modulates the deacetylation of FOXO proteins, diminishing the transcription of pro-apoptotic genes (77). Intriguingly, FOXO1, when disassociated and acetylated away from SIRT2, can complex with Autophagy Related 7 (Atg7) to trigger autophagy (78). SIRT1 is also known to augment apoptosis in cells exposed to tumor necrosis factor-alpha (TNF-α) by suppressing nuclear NF-κB activity (79). This suppression of NF-κB function is a trait shared with SIRT2 and SIRT6. In the mitochondrial environment, SIRT3, akin to SIRT1, possesses deacetylase activity that shields cells from oxidative stress-induced mortality (80). SIRT3 not only boosts the expression of Forkhead box class O3a (FoxO3A)-dependent genes but also interacts with P53 and BAG Cochaperone 2 (BAG2) to modulate growth and cellular stress response by restraining P53 activity (81). Additionally, SIRT3 plays a vital role in managing ROS levels through the modulation of superoxide dismutase 2 (SOD2), manganese dismutase, catalase, and mitochondrial isocitrate dehydrogenase (IDH2), crucial for mitigating oxidative stress-induced DNA damage, including DSBs (82–84). SIRT6, specifically, is lauded for its effectiveness in facilitating the repair of DSBs, especially under oxidative stress conditions, marking it as a key player in cellular stress management (85).

## 5 Sirtuins and their influence on aging

The impact of the Sirtuin family on mitigating cellular aging and extending lifespan has become a focal point of scientific inquiry, with particular emphasis on the roles of SIRT1 and SIRT6. Aging is categorized into replicative and premature forms, the former being attributed to telomere attrition, and the latter to the depletion of critical cellular factors such as oncogenes and tumor suppressors (86). By modulating various cellular mechanisms, Sirtuins contribute to delaying cellular senescence and extending biological longevity. This includes maintaining genomic stability, enhancing DNA repair processes, and protecting against age-related telomere degradation (87). Moreover, Sirtuins influence organismal lifespan through interactions with key regulatory pathways, including the insulin/Insulin-like growth factor-1 (IGF-1) signaling pathway and the AMP-activated protein kinase pathway (88, 89). Research highlights how pathways mediated by SIRT1, alongside Nk2 homeobox 1 (Nkx2–1), and orexin type 2 receptor (Ox2r) within the hypothalamus’ dorsal medial and lateral nuclei, can invigorate bone and muscle functions via the sympathetic nervous system, thereby fostering a physiologically youthful state that aids in delaying the aging process and prolonging life (90, 91). Furthermore, Sirt6 is actively engaged in suppressing age-associated gene expression related to cellular aging, in addition to maintaining the structural integrity of telomere chromatin, thus preventing genomic instability and cellular senescence (92).

## 6 Sirtuins’ protective role against cardiac, kidney and brain diseases

The Sirtuin family, known for its deacetylase properties, plays a significant role in safeguarding against a variety of diseases, including atherosclerotic thrombosis, hepatic steatosis, obesity induced by diet, type 2 diabetes, and myocardial infarction (93). Within the realm of cardiovascular diseases, SIRT1 stands out for its ability to mitigate atherosclerosis through the activation of endothelial nitric oxide synthase (eNOS) and the reduction of NF-κB activity in endothelial cells and macrophages (94–96). Similarly, SIRT3 has been identified as beneficial in addressing left ventricular hypertrophy, cardiomyopathy, and dyslipidemia. It achieves this by enhancing the activity of superoxide dismutase 2 (SOD2), manganese superoxide dismutase (MnSOD), and catalase (Cat), thereby preventing cardiac hypertrophy (97). Additionally, SIRT6 contributes to the improved survival of cardiomyocytes under hypoxic conditions by inhibiting necrosis or apoptosis pathways and ameliorating dyslipidemia and left ventricular hypertrophy (98).

In the context of kidney diseases, SIRT1’s presence is widespread in renal tubular cells and podocytes, where it reduces the reabsorption of epithelial sodium through interaction with methyltransferases, ultimately suppressing transcription (99). This function of SIRT1 in regulating renal sodium and water reabsorption has implications for blood pressure control. SIRT3 is pivotal in managing the mitochondrial dynamics within renal cells, regulating the microtubule network-dependent transport of functional mitochondria among renal tubular epithelial cells to maintain cellular bioenergetics (100). The role of SIRT6 in maintaining renal homeostasis has also been acknowledged (101). Moreover, the involvement of Sirtuins in neurological diseases has garnered attention in recent years, particularly given the connection between aging and Alzheimer’s disease (AD). SIRT6, by preserving telomere integrity and correcting abnormal DNA repair mechanisms, plays a role in delaying cellular senescence, which is pivotal in the context of AD (102). This multifaceted involvement of Sirtuins in disease prevention and management underscores their potential as therapeutic targets in various pathological conditions, highlighting the breadth of their influence on human health.

## 7 Sirtuins’ impact on tumorigenesis

The significance of Sirtuins in the realm of oncology has been increasingly recognized, with a growing body of evidence suggesting their involvement in regulating numerous aberrant cellular processes in cancer. These include cell cycle modulation, DNA repair mechanisms, cell survival and apoptosis, and the cellular response to genomic instability, which are pivotal in the onset and progression of cancer (**Table 2**).

**Table 2:** Sirtuins in human cancers.

Sirtuin	Human cancer type	Sirtuin levels	Role of sirtuin	References
SIRT1	Oral squamous cell carcinoma Breast cancer Liver cancer Lung cancer Gastric cancer	Reduced expression Reduced expression Increased expression Reduced expression Reduced expression	Tumour suppressor Tumour suppressor Oncogene Tumour suppressor Tumour suppressor	(101–103) (111–113) (130–132) (135–137) (141–143)
SIRT2	Gastric cancer	Increased expression	Oncogene	(144, 145)
SIRT3	Oral squamous cell carcinoma Breast cancer Liver cancer Lung cancer Gastric cancer	Increased expression Reduced expression Reduced expression Reduced expression Reduced expression	Oncogene Tumour suppressor Tumour suppressor Tumour suppressor Tumour suppressor	(104–106) (114–116) (118–120) (138, 139) (148–150)
SIRT4	Breast cancer Liver cancer	Reduced expression Reduced expression	Tumour suppressor Tumour suppressor	(117) (121–123)
SIRT5	Liver cancer Gastric cancer	Increased expression Reduced expression	Oncogene Tumour suppressor	(133) (146, 147)
SIRT6	Liver cancer Lung cancer	Reduced expression Increased expression	Tumour suppressor Oncogene	(134) (140)
SIRT7	Oral squamous cell carcinoma Breast cancer Liver cancer	Reduced expression Reduced expression Reduced expression	Tumour suppressor Tumour suppressor Tumour suppressor	(107–109) (110) (128, 129)

### 7.1 Oral cancer dynamics

The role of SIRT1 in oral cancer presents a dichotomy. Research indicates that SIRT1’s expression levels critically influence the pathophysiology of oral cancer. Stable SIRT1 expression contributes to epithelial integrity by upregulating E-cadherin expression, thus mitigating the risk of invasion and metastasis in oral cancer (103, 104). These findings suggest a tumor-suppressive role for SIRT1 in oral cancer. However, conflicting evidence exists, with some studies highlighting SIRT1’s tumor-promoting aspects. For instance, SIRT1 overexpression has been linked to cisplatin resistance in oral cancer via the upregulation of annexin A4 (105, 106). Similarly, the involvement of SIRT3 in oral cancer has been scrutinized. SIRT3 is vital for regulating cellular ROS levels, exerting anti-tumor effects in oral cancer cells by managing ROS and diminishing its activity. The absence of SIRT3 leads to increased ROS, which escalates genomic instability and fosters tumorigenesis (107–109). Furthermore, SIRT7 has been identified as a metastasis inhibitor in oral squamous cell carcinoma (OSCC), where its downregulation promotes invasion and migration of OSCC cells, whereas its overexpression hampers metastasis through the negative regulation of epithelial-mesenchymal transition (EMT) (110–112).

### 7.2 Breast cancer insights

SIRT7 has emerged as a critical modulator of the transforming growth factor-β (TGF-β) signaling pathway and an antagonist of breast cancer metastasis. It is markedly reduced in lung metastases from breast cancer in both humans and mice, indicating that a deficiency in SIRT7 propels the metastasis of breast cancer cells. The overexpression of SIRT7, particularly in early stages of breast cancer, appears crucial for countering malignancy progression (113). On the other hand, SIRT1 has been pinpointed as a key player in cell growth and survival. The miR-211–5p is known to directly target SIRT1, reducing both its gene and protein expression levels, which correlates with decreased P53 acetylation, reduced cell viability, and increased apoptosis in breast cancer cells (114–116). SIRT3, the principal mitochondrial deacetylase, safeguards key proteins by regulating mitochondrial homeostasis and antioxidants, playing a significant role in ROS production and clearance (117). A decrease in SIRT3 expression can elevate oxidative stress, leading to cell death (118). Thus, SIRT3 supports cell proliferation and survival by maintaining ROS balance and preventing apoptosis pathway activation (119). SIRT3’s role as an adjunct in breast cancer treatment, enhancing the effectiveness of chemotherapy and hormonal therapy through its diverse mechanisms, is also noteworthy. Zhang’s team showed that SIRT3 expression was higher in Tam resistant breast cancer cells. Knockdown of SIRT3 increased the sensitivity of Tam-resistant cells and induced apoptosis, with an increase in mitochondrial ROS and ERβ levels (13).In studies focusing on SIRT4, an elevated expression level in breast tissue compared to non-tumor tissues was observed, suggesting a carcinogenic role for SIRT4 at the cellular level and implicating its involvement in breast cancer development (120).

### 7.3 Hepatocellular carcinoma insights

Mitochondrial ROS production has been linked to the progression and metastasis of liver cancer, contributing to DNA damage and alterations in mitochondrial pathways. SIRT3 has been shown to counter tumor progression by suppressing HIF-1α and mitochondrial ROS production (121, 122), and by elevating SOD2 activity, a key mitochondrial antioxidant, thus bolstering resistance to oxidative stress (123). Liu et al. found that SIRT3 is underexpressed or absent in hepatocellular carcinoma (HCC) tissues (124). Compared to patients with low SIRT3 expression, those with high SIRT3 expression exhibit longer survival, better tumor differentiation, and smaller tumor size in HCC (125). Wang et al. discovered that in patients with Barcelona Clinic Liver Cancer (BCLC) stage A or without vascular invasion, SIRT3 can serve as a biomarker for post-hepatectomy recurrence (123). This suggests that SIRT3 plays a crucial role in the development of liver cancer and can serve as a reliable prognostic predictor. In HCC cells, the expression of mitochondrial Ca^2+^ uniporter (MCU) complex is upregulated, increasing mitochondrial Ca^2+^ uptake, reducing NAD^+^ levels, and decreasing NAD^+^-dependent SIRT3 deacetylase activity (126). This inhibits MnSOD activity, leading to increased ROS levels and activation of c-Jun N-terminal kinase (JNK) signaling, inducing Matrix Metallopeptidase 2 (MMP-2) activity, and promoting HCC cell migration (126). Additionally, research has shown that SIRT3 can inhibit the development and proliferation of HepG2 cells and induce apoptosis by upregulating P53 and MnSOD, increasing BCL2-Associated X (Bax) and Factor Related Apoptosis (Fas) (124). Song et al. found that SIRT3 deacetylates and activates Glycogen synthase kinase 3β (GSK-3β), inhibiting cell growth through the GSK-3β/Bax-dependent apoptotic pathway (127). Similarly, SIRT4 levels are found reduced in liver cancer cell lines and patient samples, with significant deletions at the SIRT4 locus in hepatocellular carcinoma (HCC), suggesting its protective role against liver cancer. SIRT4 impedes glutamine metabolism, enhancing ADP/AMP levels through LKB1 activation and subsequent phosphorylation of AMP-activated protein kinase (AMPK), showcasing its anticancer efficacy in liver cancer (128–130). Conversely, most liver cancer tissues exhibit elevated SIRT7 mRNA and protein levels compared to adjacent non-tumor liver tissues, playing a crucial role in negating p53-dependent cytotoxic effects in liver cancer. SIRT7’s expression, regulated by tumor suppressors miR-125a and miR-125b, underscores its upregulation in liver cancer (131, 132). SIRT1 remains consistently overexpressed in liver cancer, deemed essential at all cancer stages, with its heightened expression necessary for liver cancer persistence (133–135). SIRT5 promotes liver cancer cell proliferation; its downregulation significantly hampers this proliferation, indicating SIRT5’s role in inhibiting mitochondrial apoptosis in hepatoma cells via cytochrome c deacetylation (136). SIRT6, associated with hepatoma cell proliferation and reduced apoptosis, regulates these processes through the extracellular signal-related kinases 1 and 2 (ERK1/2) pathway, influencing the activation of endogenous apoptosis pathways (137).

### 7.4 SIRT in lung carcinoma

SIRT1’s ability to deacetylate lysine residues at core histones’ N-terminus reduces transcription by tightening DNA. It negatively regulates pro-apoptotic protein Smac and NF-κB, which delivers anti-apoptotic signals, thus protecting cells from apoptosis. SIRT1 modulates lung cancer cell apoptosis through the SIRT1-NF-κB-Smac signaling pathway (138–140). SIRT3 deacetylates the tumor suppressor Phosphatase And Tensin Homolog (PTEN), inhibiting murine double minute 2 (mdm2) transcription and stabilizing p53, yet its overexpression in non-small cell lung cancer (NSCLC) acts carcinogenically. In PTEN-deficient cancers, SIRT3’s unilateral deacetylation of p53 may contribute to NSCLC malignancies (141, 142). SIRT6, identified as a driver of NSCLC metastasis, promotes cancer cell apoptosis, suggesting its downregulation could be linked to NSCLC’s malignant progression, positioning SIRT6 as a potential therapeutic target (143).

### 7.5 SIRT in gastric cancer

SIRT1’s inhibition through NF-κB-cyclin D1 signaling plays a role in gastric cancer (GC) formation. It also impacts GC invasion and metastasis, with both *in vitro* and *in vivo* studies showing that SIRT1 reduction diminishes GC cell migration, invasion, and lung metastasis by targeting Rho GTPase Activating Protein 5 (ARHGAP5) through deacetylation and inhibition of c-JUN (144–146). SIRT2 appears to facilitate GC cell migration and invasion, possibly acting as a carcinogen in GC by activating the RAS/ERK/JNK/MMP-9 pathway and enhancing cytoplasmic PEPCK1 levels and mitochondrial activity, thus promoting cell migration and invasion. Inhibiting SIRT2 activity is crucial for mitigating GC cell migration and invasion (147, 148). Conversely, SIRT5 suppresses GC progression by inhibiting cell proliferation and glycolysis, with its downregulation or overexpression significantly impacting GC growth. SIRT5’s tumor-suppressive role in GC is mediated by its inhibition of the Oxoglutarate Dehydrogenase (OGDH) complex and interference with mitochondrial function (149, 150). A decrease in SIRT3 elevates hypoxia-inducible factor-1α (HIF-1α) expression in MGC-803 gastric cancer cells, suggesting SIRT3’s involvement in GC progression through the regulation of cellular ROS and HIF-1α stability, offering a mitochondrial tumor-suppressive mechanism (151–153).

### 7.6 Sirtuins’ diverse roles in various cancers

The Sirtuin family has been implicated in a wide range of cancers, showcasing a spectrum of expressions that influence tumor behavior. In acute myeloid leukemia (AML), heightened SIRT1 expression has been observed (154), whereas reductions in SIRT1 are linked to the onset of cancers in tissues such as the brain (glioma), bladder, ovary, and others (155–157). Prostate cancer presents a unique scenario where SIRT1 levels vary, sometimes inhibiting FOXO1 transcription and enhancing FOXO1 deacetylation to prevent apoptosis, while also capable of decreasing androgen-induced proliferation (158). Skin cancer and melanoma studies reveal a significant drop in SIRT2 expression, with mutations in SIRT2 (P199L) leading to loss of enzymatic activity and tumor proliferation. SIRT2’s knockout resulted in altered expression of keratin markers and increased stem cell markers, promoting tumor growth (159). Additionally, SIRT2 has been proposed as a tumor suppressor in glioma development, offering new molecular markers for treatment (160).

## 8 Emerging anti-tumor therapeutics targeting sirtuins

The critical role of the Sirtuin family in cancer biology has illuminated the pathway for developing novel therapeutics. This ongoing exploration into Sirtuins’ function in tumor genesis, coupled with a deeper understanding of their regulatory mechanisms, presents a promising frontier in cancer treatment with the advent of small molecule drugs targeting these enzymes (**Table 3**).

**Table 3:** Potential anti-tumor drugs targeting Sirtuins.

	Drugs	Sirtuin	Enzyme targets	References
Sirtuins activators	Resveratrol Curcumin Aspirin	SIRT1	PI3K, AKT ATM, CHK2, NF-κB Phosphor-AMPK, Phosphoacetyl CoA carboxylase	(158, 159, 161) (160) (162)
Sirtuins inhibitors	MHY2256 EX527 Salermide Sirtinol	SIRT1 SIRT2	P53 P53, P21 P53 P53	(163) (164) (165) (166)

### 8.1 Sirtuin activators: unleashing potential in cancer therapy

Resveratrol, a naturally occurring polyphenolic compound, has demonstrated significant potential in cancer prevention and treatment. As a SIRT1 activator, it enhances the protein’s expression and enzymatic action within HepG2 cells, leading to a reduction in the phosphorylation levels of the Phosphoinositide 3-Kinase (PI3K)/Protein Kinase B (PKB, AKT) pathway components. This suggests that Resveratrol’s inhibition of the PI3K/AKT pathway, a critical signaling pathway in cancer progression, is mediated through SIRT1 activation (161). Further evidence indicates that Resveratrol can impede the proliferation and migration of liver cancer cells by modulating the PI3K/AKT pathway through SIRT1’s post-translational modifications (162).

Curcumin, the primary active compound found in turmeric, exerts its anticancer effects through multiple mechanisms, including the activation of the Ataxia telangiectasia mutated (ATM)/checkpoint kinase2 (CHK2) pathway and suppression of NF-κB signaling. Research has revealed that Curcumin’s induction of DNA damage and apoptosis in tumor cells is partly facilitated by its activation of SIRT1, highlighting a dual-pathway mechanism towards exerting its anticancer effects (163).

Additional research into Resveratrol’s effects on tumor cells with constitutively active signal transducer and activator of transcription 3 (STAT3) demonstrates that this SIRT1 activator can lead to the deacetylation and subsequent inactivation of STAT3, reducing surviving gene transcription and promoting tumor cell apoptosis (164).

The role of Aspirin in cancer chemoprevention has gained attention, particularly in colorectal cancer. Studies have shown that Aspirin can induce senescence in CRC cells by upregulating SIRT1 and activating the AMPK pathway, marking a significant step forward in understanding the molecular mechanisms underlying Aspirin’s anticancer effects (165).

### 8.2 Inhibiting sirtuins: a therapeutic avenue in cancer management

The development of MHY2256, a novel SIRT inhibitor, has shown promise in reducing the viability of breast cancer cells. This compound’s ability to decrease SIRT1, 2, and 3 protein levels and increase the acetylation of p53 in MCF-7 cells signifies its potential for inducing apoptosis through p53 acetylation, offering a new therapeutic option in breast cancer treatment (166).

EX527, specifically targeting SIRT1, has been studied for its impact on glioma cell proliferation and survival. The inhibitor’s capacity to upregulate p53, acetylated p53, and the downstream target p21, thereby inducing apoptosis, presents a viable strategy for glioma therapy (167).

Salermide, a reverse amide compound, exhibits potent inhibitory effects against SIRT1 and SIRT2 *in vitro*. Its unique ability to induce p53-mediated apoptosis in breast cancer cells without affecting normal cells underscores its therapeutic potential (168).

A comprehensive analysis comparing Sirtinol, Salermide, and EX527 revealed their effectiveness as SIRT1/2 inhibitors, with EX527 showing the highest specificity towards SIRT1. This comparative study suggests the necessity of targeting both SIRT1 and SIRT2 to achieve optimal pro-apoptotic effects in cancer therapy (169).

### 8.3 MicroRNAs: integrating into sirtuin-targeted therapies

The role of microRNAs (miRNAs) as key regulators in cancer has gained prominence, with their dual capacity to act as either oncogenes or tumor suppressors. This dual functionality allows miRNAs to finely tune the expression of crucial genes involved in tumorigenesis, including those related to the Sirtuin family, thereby offering a nuanced approach to cancer therapy (**Table 4**).

**Table 4:** MicroRNAs that are associated with human cancers.

MiRNA	Sirtuin	Cancer	References
MiR-34a	SIRT1	Prostate cancer	(167, 168)
MiR-204-5p	SIRT1	Liver cancer	(169, 170)
MiR-22	SIRT1	Glioblastoma	(169, 170)
MiR-217	SIRT1	Pancreatic cancer	(171)
MiR-125a-5p	SIRT7	Lung cancer	(172)

One pivotal example is MicroRNA-34a (miR-34a), which is activated by p53 and targets SIRT1 along with other critical oncogenes. The modulation of SIRT1 by miR-34a leads to cell cycle arrest and apoptosis, making miR-34a a potential ally in chemotherapy regimens aimed at enhancing cancer cell susceptibility to treatment. This synergy between miRNA-34a and chemotherapy could lead to more effective therapeutic strategies, enhancing treatment efficacy while minimizing side effects (170, 171).

Further, the miRNAs miRNA-204–5p and miR-22 have been identified as potent regulators of SIRT1, showcasing the capability of miRNAs to inhibit cancer progression by modulating key molecular pathways. These miRNAs can suppress SIRT1 activity, influencing cellular processes such as cell survival, proliferation, and apoptosis. By altering SIRT1 levels, miRNA-204–5p and miR-22 can potentially reshape the tumor microenvironment, thereby stalling cancer progression and providing new avenues for targeted cancer therapies (172, 173). Furthermore, miRNA-217 could target SIRT1 to induce epithelial-mesenchymal transition (EMT), and lead to poor prognosis of pancreatic cancer (174). MiR-125a-5p has been reported to increase radioresistance in NSCLC via upregulation of SIRT7 (175). The expanding research into miRNAs offers promising insights into their potential to manipulate Sirtuin activity, revealing a complex network of genetic regulation that could be harnessed to develop innovative cancer treatments. These findings underscore the need for continued exploration into miRNA-mediated pathways as integral components of cancer therapeutics, where the strategic targeting of Sirtuins could lead to groundbreaking advancements in cancer treatment.

## 9 Conclusion and perspectives

The multifaceted roles of Sirtuins in cancer biology have become increasingly evident, revealing their profound influence on tumorigenesis and cancer progression through diverse mechanisms. These proteins, with their capacity to either suppress or promote tumor growth, embody the complex nature of cancer regulation at the molecular level. While Sirtuins can act as guardians by inhibiting tumor development, they can also play a role in facilitating cancer progression under certain conditions. This dual functionality underscores the nuanced understanding required to harness their potential in cancer therapy effectively. Despite the strides made in elucidating the functions of Sirtuins in cancer, numerous questions remain. The intricate balance between their protective and oncogenic roles presents a significant challenge in developing therapeutic strategies. However, the growing body of evidence underscores the significant impact these enzymes have on the cancer landscape. Their ability to modulate key cancer pathways offers a promising avenue for targeted therapy, making them critical components in the fight against cancer.

Moreover, the potential of Sirtuins to synergize with existing anticancer drugs opens new possibilities for combination therapies. By integrating Sirtuin-targeted treatments with conventional chemotherapy, targeted therapy, or immunotherapy, we can potentially enhance therapeutic efficacy and overcome resistance mechanisms. This approach not only amplifies the anti-tumor effects but also provides a strategy to mitigate the adverse side effects associated with high-dose monotherapies. As research continues to advance, it is anticipated that novel Sirtuin modulators will emerge, offering more precise and personalized treatment options for cancer patients. The development of these modulators, guided by an in-depth understanding of Sirtuin biology and cancer-specific contexts, could lead to breakthroughs in cancer care. Furthermore, exploring the interplay between Sirtuins and the tumor microenvironment may unveil additional therapeutic targets and strategies to prevent tumor metastasis and recurrence.

In conclusion, Sirtuins represent a pivotal frontier in cancer research, with the potential to revolutionize cancer treatment paradigms. Their dual roles in cancer biology reflect the complexity of targeting these enzymes but also highlight the immense therapeutic potential they hold. As we continue to unravel the intricacies of Sirtuin function in cancer, the future holds promise for innovative therapies that could significantly impact patient outcomes in the battle against cancer.

## Author contributions

LY: Writing – original draft. YL: Writing – original draft. SS: Writing – original draft. YZ: Writing – original draft. YipW: Funding acquisition, Writing – original draft. HW: Funding acquisition, Writing – original draft. ZY: Funding acquisition, Writing – review & editing. YiW: Conceptualization, Funding acquisition, Investigation, Project administration, Writing – review & editing.
